# Health system implementation of the PROMIS Cognitive Function Screener in the Medicare Annual Wellness Visit: framing as abilities versus concerns

**DOI:** 10.1186/s41687-024-00699-8

**Published:** 2024-04-10

**Authors:** Jordan M. Harrison, Natalie C. Ernecoff, Jin-Shei Lai, Janel Hanmer, Rebecca Weir, Anthony Rodriguez, Michelle M. Langer, Maria O. Edelen

**Affiliations:** 1 RAND Corporation, 4570 Fifth Avenue #600, 15213 Pittsburgh, PA USA; 2 Northwestern University Feinberg School of Medicine, Chicago, IL USA; 3 Department of General Internal Medicine, University of Pittsburgh, Pittsburgh, PA USA; 4 RAND Corporation, Arlington, VA USA; 5 RAND Corporation, Boston, MA USA; 6 Brigham and Women’s Hospital, Boston, MA USA

**Keywords:** Alzheimer’s disease, Dementia, Cognition, Cognitive function, Cognitive impairment, Screening, Medicare, Primary care

## Abstract

**Background:**

Cognitive assessment is a required component of the Medicare Annual Wellness Visit (AWV). In this prospective study, we evaluated acceptability and usefulness of a patient-reported outcome measure (the PROMIS® Cognitive Function Screener, or PRO-CS) to screen for cognitive impairment during the AWV. We compared two versions of the PRO-CS: Abilities and Concerns.

**Methods:**

We developed PRO-CS Abilities and PRO-CS Concerns using items from the PROMIS Cognitive Function item banks. We partnered with a large health system in Pennsylvania to implement an electronic health record (EHR)-integrated version of the 4-item PRO-CS into their AWV workflow. PRO-CS Abilities was implemented in June 2022 and then replaced with PRO-CS Concerns in October 2022. We used EHR data to evaluate scores on Abilities versus Concerns and their association with patient characteristics. We gathered feedback from providers on experiences with the PRO-CS and conducted cognitive interviews with patients to evaluate their preferences for Abilities versus Concerns.

**Results:**

Between June 2022 and January 2023, 3,088 patients completed PRO-CS Abilities and 2,614 patients completed PRO-CS Concerns. Mean T-scores for Abilities (54.8) were slightly higher (indicating better cognition) than for Concerns (52.6). 10% of scores on Abilities and 13% of scores on Concerns indicated concern for cognitive impairment (T-score < 45). Both Abilities and Concerns were associated with clinical characteristics as hypothesized, with lower scores for patients with cognitive impairment diagnoses and those requiring assistance with instrumental activities of daily living. Abilities and Concerns had similar negative correlations with depression (*r*= -0.31 versus *r*= -0.33) and anxiety (*r*= -0.28 for both), while Abilities had a slightly stronger positive correlation with self-rated health (*r* = 0.34 versus *r* = 0.28). In interviews, providers commented that the PRO-CS could be useful to facilitate conversations about cognition, though several providers noted potential limitations of patient self-report. Feedback from patients indicated a preference for PRO-CS Concerns.

**Conclusions:**

Our findings suggest potential utility of the PRO-CS for cognitive screening in the Medicare AWV. PRO-CS Abilities and Concerns had similar associations with patient clinical characteristics, but the Concerns version was more acceptable to patients.

**Supplementary Information:**

The online version contains supplementary material available at 10.1186/s41687-024-00699-8.

## Abstract (de)

One of the barriers to effective cognitive screening during the Medicare Annual Wellness Visit (AWV) is the lack of a brief, reliable, and validated screening tool for cognitive impairment that can be easily integrated into the electronic health record. Use of a patient-reported outcome measure for this purpose could alert clinicians to changes in cognition that require further assessment. Previous research has demonstrated that patient-reported cognitive abilities are potentially a separate construct from patient-reported cognitive concerns, and it is unclear which construct is optimal for use in a cognitive screener. In this prospective study, we evaluated acceptability and usefulness of a patient-reported outcome measure (the PROMIS® Cognitive Function Screener, or PRO-CS) to screen for cognitive impairment during the AWV. We compared two versions of the PRO-CS: Abilities and Concerns. Our findings suggest the PRO-CS was acceptable to providers and patients, though some providers had reservations about the use of patient self-report for cognitive screening. PRO-CS Abilities and PRO-CS Concerns had similar associations with patient clinical characteristics, but feedback from patients and experts indicated a preference for PRO-CS Concerns. Given the time and resource constraints in the Medicare AWV, our findings suggest the PRO-CS may be useful as the first step to engage providers, patients, and families in conversations about cognition. Further research is needed to understand the impact of the PRO-CS on patient-provider communication, as well as subsequent diagnostic testing and referrals.

## Background

The Medicare Annual Wellness Visit (AWV) is a yearly primary care visit focused on preventive care and screening. Individuals who receive medical coverage through Medicare (including beneficiaries aged 65 and older and those with certain disabilities or health conditions) have the option to schedule an AWV with their primary care provider, such as a physician or nurse practitioner, to review their health risk factors and develop a personalized prevention plan. Cognitive assessment is a required component of the AWV, yet clinician approaches to this assessment vary widely [1, 2]. Many clinicians rely on informal observation or input from families, which is subject to bias and likely ineffective for early detection of cognitive impairment [1, 2]. An initial standardized cognitive assessment should provide either a baseline for cognitive surveillance or a trigger for further evaluation [2]. However, limitations of existing assessments include the time and burden to administer and lack of sensitivity for detection of mild cognitive impairment. One of the barriers to effective cognitive assessment during the AWV is the lack of a brief, reliable, and validated screening tool for cognitive impairment that can be patient administered and easily integrated into the electronic health record (EHR). Use of a patient-reported outcome measure for this purpose could alert clinicians to changes in cognition that require further assessment.

The Patient Reported Outcome Measurement Information System (PROMIS)® Cognition measurement domain is represented by two co-calibrated item banks that consist of items developed to assess cognitive function perceived by individuals [3, 4]. The PROMIS item banking approach has the advantages of being flexible, efficient, precise, and highly suitable for integration into electronic administration platforms, including EHRs. The items are available in several languages and have been tested for bias according to demographic characteristics including race/ethnicity and age [3–8]. Further, comprehensive content coverage of items in the bank allows for tailored selection of optimal items for screening purposes. These properties make the PROMIS Cognition an ideal source for developing a patient-administered screener for cognitive impairment that could be used in the Medicare AWV. The PROMIS Cognition includes two separate but co-calibrated item banks: Cognitive Function– Abilities (cognitive abilities framing) and Cognitive Function (cognitive concerns framing). Previous research has demonstrated that patient-reported cognitive abilities are potentially a separate construct from patient-reported cognitive concerns [3]. It is unclear which construct—cognitive abilities or cognitive concerns—is optimal for use in a patient-reported cognitive screener.

In this prospective study, we evaluated acceptability and usefulness of a patient-reported outcome measure (the PROMIS Cognitive Function Screener, or PRO-CS) to screen for cognitive impairment during the Medicare AWV. We compared two versions of the PRO-CS: Abilities and Concerns, developed from the PROMIS Cognitive Function– Abilities and PROMIS Cognitive Function item banks, respectively.

## Methods

All study procedures were approved by the RAND Human Subjects Protection Committee.

### Development of the PRO-CS

We gathered input from community members, caregivers, providers, and experts to identify content areas most important to include in a screening tool (reported in detail elsewhere) [9]. We identified four content areas deemed self-reportable, applicable to most patients, and associated with early cognitive impairment: memory/general function, multi-tasking, working memory, and verbal fluency. With input from an advisory group of clinical and research experts in cognitive impairment and dementia, we selected four-item sets from the PROMIS Cognitive Function.

Abilities item bank to assess each of these domains. Subsequently, in response to provider feedback, we selected a comparable set of items from the PROMIS Cognitive Function item bank (concerns framing) that covered the same domains.

The PRO-CS Abilities and PRO-CS Concerns each consist of four items to assess patient-perceived cognitive function in the past seven days (Tables 1 and 2, respectively). The Abilities and Concerns items have different response scales. The Abilities items assess *intensity* on a 5-point scale (Not at all; A little bit; Somewhat; Quite a bit; Very much), while the Concerns items assess *frequency* on a 5-point scale (Never; Rarely; Sometimes; Often; Very often) [4].

**Table 1:** PRO-CS cognitive abilities: response frequencies

	Number and percent of responses (total *N* = 3,088)					
In the past 7 days…	(1) Not at all	(2) A little bit	(3) Some-what	(4) Quite a bit	(5) Very much	Missing
My memory has been as good as usual	69 (2.3%)	123 (4.0%)	409 (13.4%)	1,161 (38.0%)	1,293 (42.3%)	33 (1.1%)
I have been able to keep track of what I am doing, even if I am interrupted	54 (1.8%)	85 (2.8%)	275 (9.0%)	1,025 (33.7%)	1,607 (52.8%)	42 (1.4%)
I have been able to learn new things easily, like telephone numbers or instructions	70 (2.3%)	127 (4.2%)	389 (12.8%)	1,091 (35.8%)	1,367 (44.9%)	44 (1.4%)
I have been able to bring to mind words that I wanted to use while talking to someone	33 (1.1%)	121 (4.0%)	464 (15.2%)	1,364 (44.6%)	1,075 (35.2%)	31 (1.0%)

**Table 2:** PRO-CS cognitive concerns: response frequencies

	Number and percent of responses (total *N* = 2,614)					
In the past 7 days…	(5) Never	(4) Rarely (Once)	(3) Some-times (Two or three times)	(2) Often (About once a day)	(1) Very often (Several times a day)	Missing
I have had trouble remembering whether I did things I was supposed to do, like taking a medicine or buying something I needed	1,133 (43.7%)	1,083 (41.8%)	310 (12.0%)	42 (1.6%)	24 (0.9%)	22 (0.8%)
I have had trouble shifting back and forth between different activities that require thinking	1,810 (70.1%)	600 (23.3%)	129 (5.0%)	19 (0.7%)	23 (0.9%)	33 (1.3%)
I have had trouble remembering new information, like phone numbers or simple instructions	1,325 (51.2%)	846 (32.7%)	332 (12.8%)	52 (2.0%)	31 (1.2%)	28 (1.1%)
I have had trouble recalling the name of an object while talking to someone	997 (38.5%)	1,004 (38.7%)	501 (19.3%)	60 (2.3%)	31 (1.2%)	21 (0.8%)

### Implementation of the PRO-CS

We partnered with a large health system in Pennsylvania to implement an EHR-integrated version of the PRO-CS in their Medicare AWV workflow. This change was implemented as part of the rollout of a new AWV questionnaire across the entire health system. Patients complete the AWV questionnaire either online prior to the visit or on paper when they arrive for the visit. Among other updates to the questionnaire, the health system added the PRO-CS Abilities items to replace the previous cognitive screening items. The new AWV questionnaire was implemented system-wide in June 2022. After initially implementing the Abilities version of the PRO-CS, the health system switched to the Concerns version in October 2022.

### Electronic health record data

We extracted EHR data for all patients with AWVs who completed electronically administered AWV questionnaires between 6/6/22 and 1/19/23. Data for patients who completed paper questionnaires was not available in the EHR. We identified responses to both versions of the PRO-CS and other patient-reported information from the AWV questionnaire, including instrumental activities of daily living (IADL) performance, self-rated health (scale of 1–5 where 1 = poor and 5 = excellent), and scores on the Patient Health Questionnaire-2 (PHQ-2, two items to assess depression symptoms, resulting in a score of 0–6) [10] and General Anxiety Disorder-2 (GAD-2, two items to assess anxiety symptoms, resulting in a score of 0–6) [11]. Higher scores on the PHQ-2 and GAD-2 indicate more depression or anxiety, respectively.

To identify patients with diagnoses associated with cognitive impairment, we identified International Classification of Diseases (ICD)-10 codes for diagnoses reported in the EHR and used the Clinical Classifications Software Refined (CCS-R) to group patients’ diagnoses into clinically meaningful diagnostic groups. Two physicians from our team reviewed all CCS-R categories under “neurologic disorders” and classified them as not associated with cognitive impairment, possibly associated with cognitive impairment, or definitely associated with cognitive impairment. We used this system to classify each patient as having no cognitive impairment, possible cognitive impairment, or definite cognitive impairment based on their ICD-10 diagnoses [12].

### Statistical analyses

The PROMIS items use item response theory (IRT)-based scoring [13]. We transformed PRO-CS Abilities and Concerns raw scores into the T-score metric (mean 50, standard deviation 10) using PROMIS item parameters. Higher scores indicate better cognition for both PRO-CS versions. Cognitive impairment is defined as a T-score less than 45 (0.5 standard deviations below the population mean of 50) [14].

We used descriptive statistics to compare scores on PRO-CS Abilities and Concerns. We used t-tests and analysis of variance (ANOVA) to evaluate the association of PRO-CS scores with patient clinical characteristics (IADLs and cognitive impairment diagnoses) and Pearson’s correlation to evaluate their association with other patient-reported information (self-rated health, depression, and anxiety).

### Qualitative feedback

To gather feedback on acceptability and usefulness of the PRO-CS, we conducted interviews with a purposive sample of primary care providers from the health system who regularly conducted AWVs (*n* = 8), including several providers involved in the health system’s AWV working group. We interviewed providers in August 2022, prior to the switch to PRO-CS Concerns. Subsequently, we conducted cognitive interviews with primary care patients aged 65 and older from the same health system (*n* = 10) between November 2022 and March 2023 to evaluate their preferences for PRO-CS Abilities versus Concerns. Provider and patient interviews were analyzed using rapid qualitative analysis of post-interview notes to identify emergent themes [15]. We entered information directly into summary templates organized by topic (e.g., for provider interviews: initial perceptions of the PRO-CS, impact on workflow, usefulness, acceptability to patients; for patient interviews: general impressions of the items, ease of interpretation, abilities versus concerns framing, response scales). Data were analyzed concurrently with data collection to identify areas for additional probing and to identify when data saturation was reached (i.e., when no new themes emerged).

Finally, we presented our qualitative and quantitative findings to our project advisory group and solicited their feedback regarding usefulness of PRO-CS Abilities versus Concerns for cognitive screening in the AWV.

## Results

### EHR-based outcomes

Patients who completed the PRO-CS electronically prior to their AWV (*n* = 5,702) were predominantly female (58%), White (96%), and non-Hispanic (99.6%). The majority of patients (66%) were 65 to 74 years of age, and the rest were 75 and older (34%). Most patients (87%) had no diagnoses associated with cognitive impairment, 11% had possible cognitive impairment, and 2% had definite cognitive impairment. A total of 3,088 AWV patients completed the Abilities version of the PRO-CS (Table 1) and 2,614 patients completed the Concerns version (Table 2). As shown in Table 3, mean T-scores for Abilities were slightly higher than for Concerns (54.8 versus 52.6), and the range of scores was higher for Abilities than for Concerns (25.1–65.4 versus 23.2–62.0). 10% of scores on Abilities and 13% of scores on Concerns indicated concern for cognitive impairment (T-score < 45).

**Table 3:** PRO-CS cognitive abilities versus cognitive concerns: descriptive statistics and association with patient characteristics

	PROMIS Cognitive Abilities (*N* = 3,088)	PROMIS Cognitive Concerns (*N* = 2,614)
Mean (SD) T-score	54.8 (8.0)	52.6 (7.1)
Min, Max T-score	25.1, 65.4	23.2, 62.0
T-Score indicates concern for cognitive impairment (> 0.5 SD below mean), n (%)	316 (10.2%)	343 (13.1%)
***Mean (SD) PRO-CS score according to patient clinical characteristics***		
Requires assistance with one or more instrumental activities of daily living Yes No	48.5 (9.2) 55.6 (7.5)	47.3 (9.0) 53.2 (6.6)
Cognitive impairment (CI)* No CI diagnosis Possible CI diagnosis Definite CI diagnosis	55.3 (7.7) 52.9 (8.8) 43.5 (8.6)	52.8 (6.9) 51.7 (7.2) 42.6 (11.5)
***Correlation of PRO-CS score with other patient-reported information (Pearson’s r)***		
Self-rated health (scale of 1–5 where 1 = poor and 5 = excellent)	0.34	0.28
Patient Health Questionnaire-2 (depression)	-0.31	-0.33
General Anxiety Disorder-2 (anxiety)	-0.28	-0.28

*Categories are based on ICD-10 diagnoses

PRO-CS Abilities and Concerns were associated with clinical characteristics as hypothesized, with lower (more impaired) scores for patients with cognitive impairment diagnoses (F = 72.50 for Abilities, F = 44.13 for Concerns, *p* < 0.001 for both) and those requiring assistance with IADLs (t = 13.52 for Abilities, t = 10.45 for Concerns, *p* < 0.001 for both). Both framings of PRO-CS scores were positively correlated with self-rated health and negatively correlated with depression and anxiety (*p* < 0.001 for all). Abilities had a slightly stronger correlation with self-rated health (*r* = 0.34 versus *r* = 0.28), while Concerns and Abilities had similar correlations with depression (*r* = -0.33 versus *r* = -0.31) and the same correlation with anxiety (*r* = -0.28) (Table 3). The PHQ-2 and GAD-2 had good internal consistency reliability (α = 0.81 for both), comparable to that reported in the literature [16].

### Acceptability and feasibility

#### Providers

In interviews, providers reported mixed feedback about usefulness of PRO-CS to screen for cognitive impairment during the AWV. Several providers commented that the PRO-CS was useful to facilitate conversations with patients about cognition using examples from their daily lives, and a lower (more impaired) PRO-CS score may prompt them to conduct a performance-based test such as the Montreal Cognitive Assessment (MoCA). As one provider stated:

We still use the old Mini-Cog. Looking at these [PRO-CS items], they are more practical in terms of day-to-dayfunction… they get to the heart of what people need to do cognitively to function…if patients had particular issues, it would be worth investigating further. Can you give me examples of situations when you lose track of what you’re doing?

Providers also noted the potential limitations of a patient-reported cognitive screener. Several providers commented that some patients may not recognize when they are experiencing signs of cognitive impairment, and some patients are reluctant to report concerns about memory or cognition. As one provider commented:

Certainly patient perception is important, though you wonder if the patient is under-reporting problems, so you worry about that. Whereas if they have to demonstrate to you with the MoCA, it’s actually testing them…that would be the biggest difference in my mind.

Several providers had concerns about false positives, as some patients are “forgetful” but don’t have true cognitive impairment. One provider preferred to use a screener such as the Mini-Cog with a high negative predictive value, as opposed to a screener designed to detect early signs of cognitive impairment. When asked about patient-level barriers to self-reported cognitive screening, several providers commented that their AWV patients with lower education level, low literacy, limited English proficiency, or those in the oldest age groups may struggle to complete the PRO-CS. Overall, providers felt the PRO-CS would work better for younger AWV patients and those with higher education level. When asked about the potential utility of interpretive guidance, nearly all providers commented that interpretive guidance would make the PRO-CS more actionable. Several providers requested guidance for appropriate diagnostic testing and referrals according to ranges of scores on the PRO-CS.

We received feedback from several providers that the Abilities framing of the PRO-CS was confusing to patients. Providers reported that it was unclear to patients which responses were “positive” and which were “negative” (i.e., indicative of cognitive impairment). As one provider stated:

*There were some double negatives [on PRO-CS Abilities] that made it confusing. If I’m doing okay, which answer do I put? “1” is a good score, “5” is a bad score… there was confusion about that.*

Providers noted that other assessments in the AWV questionnaire are oriented around concerns and problems (e.g., symptom burden, functional impairment). The response scale for PRO-CS Abilities (in which higher responses on the scale indicate *better* cognition) did not align with the response scales for these other assessments. Based on similar feedback vetted by the health system’s AWV working group, the health system switched to PRO-CS Concerns in October 2022 to better align with other assessments in the AWV questionnaire.

#### Patients

Ten primary care patients aged 65 and older (50% female; mean age 71 years; 60% Non-Hispanic White; 40% Non-Hispanic Black) participated in cognitive interviews to understand their preferences for PRO-CS Abilities versus Concerns. Seven patients expressed a preference for Concerns, and three did not have a strong preference for one version over the other. Patients’ preference for Concerns versus Abilities was driven in part by the response scales. Most patients found the frequency-based response options for the Concerns items easier to understand than the intensity-based response options for Abilities. Several patients commented that the distinction between different response options for the Abilities items was unclear; for example, the difference between “quite a bit” and “very much” or between “a little bit” and “somewhat” was unclear. Notably, three patients initially misinterpreted the Abilities items, answering with the opposite response they intended (e.g., the patient had no cognitive concerns and responded “1 - not at all” instead of “5 - very much”). Several patients commented that it was easier to answer questions about cognitive concerns as opposed to cognitive abilities, and the Concerns framing was more consistent with typical surveys about symptoms administered during primary care visits. As one patient stated:

[The Concerns framing] is simpler and more direct. It’s easier to say, ‘I haven’t had trouble,’ rather than think about all the things you’re able to do.

### Expert advisors

Four experts from our project advisory group provided feedback on our findings following implementation of the PRO-CS. Overall, advisors agreed that the PRO-CS Concerns was preferable for cognitive screening in the AWV compared to the PRO-CS Abilities because the purpose of the screener is to assess whether patients perceive a problem. One advisor noted the caveat that some patients do not have insight into their own cognitive functioning, therefore there is no gold standard cognitive screener that will work for all populations, and patient-reported measures do not provide the same information as performance-based measures. The advisor noted, however, that a patient-reported cognitive screener can serve as an “ice breaker” to facilitate more in-depth discussions with patients and families regarding cognitive concerns. Another advisor commented that patient-reported measures, along with input from family members or caregivers, may provide a better early indication of cognitive impairment than performance-based measures. Advisors also noted the importance of actionability and recommended further research to assess whether scores on the PRO-CS impact clinician behavior including subsequent diagnostic testing and referrals, as well as research to explore the impact of the PRO-CS on providers’ communication with patients and family members about cognitive concerns.

## Discussion

Our findings suggest potential utility of the PRO-CS for cognitive screening in the Medicare AWV. The PRO-CS was acceptable to providers and patients, though some providers had reservations about the use of patient self-report for cognitive screening. PRO-CS Abilities and PRO-CS Concerns had similar associations with cognitive impairment diagnoses, IADLs, and other patient-reported information such as depression and anxiety, though Abilities had a stronger correlation with self-rated health. However, feedback from patients and experts indicated a preference for PRO-CS Concerns. Overall, patients found the Concerns framing more intuitive than the Abilities framing. Notably, they found the frequency-based response scale for Concerns easier to interpret than the intensity-based response scale for Abilities.

In previous studies, patient-reported cognitive dysfunction has been correlated with measures of emotional distress such as anxiety and depression, suggesting that patients with negative affect may endorse greater cognitive dysfunction [17]. The PROMIS Cognitive Function Abilities items could, in theory, reduce bias associated with negative emotional states by assessing capabilities as opposed to deficits [4]. In a previous study of older adults at risk for cognitive decline, PROMIS Cognitive Function Concerns items had a stronger correlation with depression and activities of daily living, while Abilities items had a stronger correlation with objective measures of cognition [8]. In contrast, we found that PRO-CS Abilities and Concerns had very similar correlations with depression and the same correlation with anxiety.

Use of a patient-reported measure for cognitive screening has advantages as well as limitations. Flexibility in timing and mode of administration is a major advantage of patient-reported measures [4, 8]. The PRO-CS can be completed prior to patient visits, unlike many performance-based measures such as the MoCA which require time for providers to administer. A drawback to patient self-report is that not all patients recognize issues with memory or cognition, and some patients may be reluctant to report these issues to their provider. When possible, soliciting input from a family member about the patient’s cognition can provide useful information. Notably, patient-reported measures may not align with performance-based measures. Some evidence suggests patient self-report is only moderately correlated with performance-based measures of cognition such as the MoCA [18, 19]. However, the PRO-CS provides information about patients’ everyday functioning that may be particularly useful for early detection of cognitive impairment. A patient-reported cognitive screener can serve as an “ice breaker” to promote communication between providers and patients about cognitive concerns. Interpretive guidance for providers may facilitate appropriate follow-up such as diagnostic testing and referrals for patients whose scores indicate concern for cognitive impairment.

### Limitations

Our study had some limitations. Our data are limited to a single health system, and patients who completed the PRO-CS were predominantly Non-Hispanic White. We did not have data on patient education level or literacy, which may impact responses to the PRO-CS items [7]. We did not have performance-based cognitive assessments to compare with scores on the PRO-CS. In addition, our quantitative analyses were limited to patients who completed the AWV questionnaire online prior to their visit. These patients likely differ in some ways from patients who completed the questionnaire on paper.

## Conclusions

Given the time and resource constraints in the Medicare AWV, an extremely brief, patient-reported cognitive screener such as the PRO-CS may be useful as the first step to engage providers, patients, and families in conversations about cognition. PRO-CS Abilities and Concerns had similar associations with patient characteristics, but the Concerns version was more acceptable to patients. Further research is needed to understand the impact of the PRO-CS on patient-provider communication, as well as subsequent diagnostic testing and referrals.

### Electronic supplementary material

Below is the link to the electronic supplementary material.

Supplementary Material 1

## Data Availability

The datasets generated and/or analyzed during the current study are not publicly available because they are protected health information from the electronic health record. However, an agreement to share a deidentified analytic dataset may be negotiated by contacting the study PI.
