# Supplementary material for: Health system implementation of the PROMIS Cognitive Function Screener in the Medicare Annual Wellness Visit: framing as abilities versus concerns
Source: J Patient Rep Outcomes. 2024 Apr 10;8:43. doi: 10.1186/s41687-024-00699-8 (PMC11006629; doi:10.1186/s41687-024-00699-8)
Supplement: Supplementary file 1 — Supplementary Material 1 [file 41687_2024_699_MOESM1_ESM.docx]

**Declarations**

**Ethics approval and consent to participate:** All study procedures were approved by the lead author’s Human Subjects Protection Committee.

**Consent for publication:** Not applicable.

**Availability of data and materials:** The datasets generated and/or analyzed during the current study are not publicly available because they are protected health information from the electronic health record. However, an agreement to share a deidentified analytic dataset may be negotiated by contacting the study PI (medelen@bwh.harvard.edu).

**Competing interests:** The authors have no competing interests to disclose.

**Funding:** This work was supported by funding from the National Institute on Aging (R61AG069776).

**Authors' contributions:** All authors contributed to study design, conceptualization, interpretation of results, and text revision. JMH drafted the text and RW conducted the analysis.

**Acknowledgements:** None.
